# AC3^®^ exerts cytotoxic and anti-migratory activity and modulates the gene expression of TNF-α, inflammatory mediators, and components of the CD39/CD73/adenosine axis in cutaneous melanoma cell lines

**DOI:** 10.1007/s12032-026-03321-7

**Published:** 2026-07-15

**Authors:** Daiane Manica, Gilnei Bruno da Silva, Bruna Cristina Ozelame, Vitória Capelli de Mello, Vitor Asmann da Rosa, Caroline Ortmann, Isabella Del Bosco Brunetti de Camargo, Anju Majeed, Shaheen Majeed, Aniela Pinto Kempka, Ariane Zamoner, Margarete Dulce Bagatini

**Affiliations:** 1 Postgraduate Program in Biochemistry, Federal University of Santa Catarina, Florianópolis, SC Brazil; 2 Multicentric Postgraduate Program in Biochemistry and Molecular Biology, State University of Santa Catarina, Lages, SC Brazil; 3 Postgraduate Program in Health Sciences, Community University of the Chapecó Region (Unochapecó), Chapecó, SC Brazil; 4 Postgraduate Program in Biomedical Sciences, Federal University of Fronteira Sul, Chapecó, SC Brazil; 5 Sabinsa Brasil, Florianópolis, Santa Catarina Brazil; 6 Sami-Sabinsa Group, Bangalore, Karnataka India; 7 Sabinsa Corporation, Payson, UT USA; 8 LaBioSignal - Laboratory of Biochemistry and Cell Signal, Department of Biochemistry, Federal University of Santa Catarina, Florianópolis, SC Brazil; 9 Federal University of Southern Frontier , SC 484, Km 02, Southern Frontier, Chapecó, SC 89812000 Brazil

**Keywords:** Curcuminoids, Cutaneous melanoma, Apoptosis, TNF/TNFR1 axis, Purinergic signaling, Antitumor therapy

## Abstract

Cutaneous melanoma (CM) poses a critical public health concern due to its high recurrence rates and resistance to standard treatments. This study investigated the antineoplastic potential of *Curcuma Longa L.* extract enriched with Bisdemethoxycurcumin (AC3^®^) on human melanoma cell lines A375 and SK-MEL-28. The A375 and SK-MEL-28 cell lines were cultured according to the manufacturer’s recommendations and were subsequently treated with different concentrations of AC3^®^. The MTT test and fluorescence microscopy were used to assess cell viability. The wound-healing assay was used to assess cell migration following compound treatment. To determine reactive oxygen species (ROS) levels, we used the fluorescence assay with 2,7-dichlorodihydrofluorescein diacetate. Subsequently, the molecular pathways associated with apoptosis were evaluated using RT-PCR, including modulation of the inflammatory profile and purinergic signaling components. AC3^®^ effectively inhibited cell proliferation and migration in both melanoma cell lines. In A375 cells, the compound reduced ROS levels and significantly modulated apoptotic pathways, including upregulation of caspase-3 expression. Similarly, in SK-MEL-28 cells, increased expression of caspase-8 and caspase-3 indicated the activation of both extrinsic and intrinsic apoptotic mechanisms at the transcriptional level. Additionally, AC3^®^ influenced the inflammatory response by increasing the expression of Tumor Necrosis Factor Alpha (TNF-α), Interleukin 6 (IL-6), and NLR Family Pyrin Domain Containing 3 (NLRP3) in A375 cells while downregulating IL-6 and NLRP3 expression in SK-MEL-28 cells, highlighting its effect on processes related to tumor development and progression. Furthermore, the compound modulates the expression and enzymatic activity of ectonucleoside triphosphate diphosphohydrolase 1 (CD39) and ecto-5’-nucleotidase (CD73), key regulators of purinergic signaling, suggesting its potential to affect immune evasion mechanisms in melanoma. These findings position the AC3^®^ compound as promising for cutaneous melanoma by targeting critical pathways involved in tumor progression and cell survival.

**Graphical abstract:**

**Figure:**
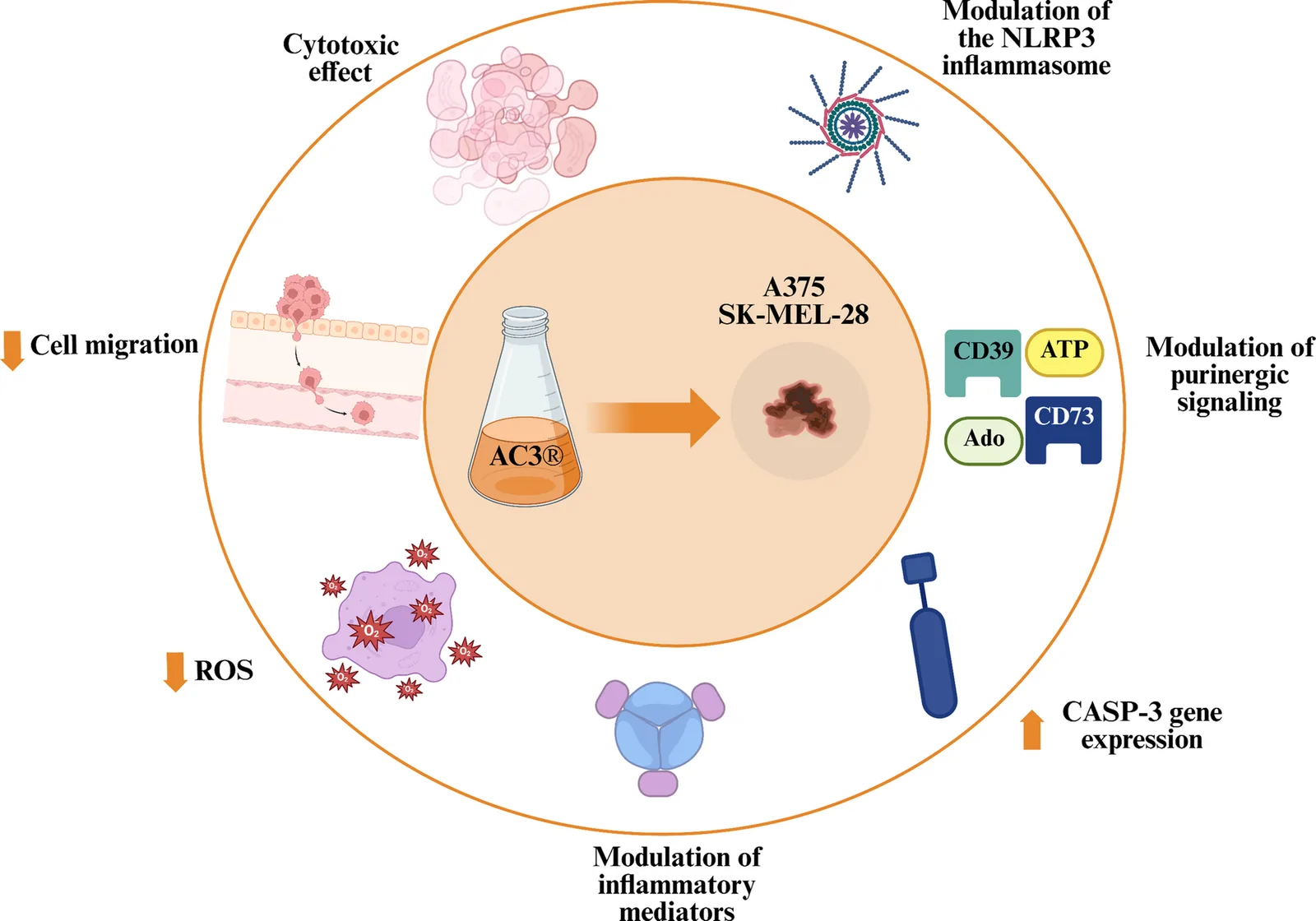
Cytotoxic effect of AC3® in metastatic cutaneous melanoma cells - In our study, we demonstrated that AC3® reduces the viability, migration, and reactive oxygen species (ROS) levels in the metastatic cutaneous melanoma cell lines A375 and SK-MEL-28. In molecular biology assays, we observed increased gene expression of tumor necrosis factor alpha (TNF) and Caspase-3 (CASP-3). Furthermore, we observed that the extract can modulate purinergic signaling, thereby reducing immunosuppression.

**Supplementary Information:**

The online version contains supplementary material available at https://doi.org/10.1007/s12032-026-03321-7.

## Introduction

Since ancient times, humans have used natural compounds to prevent and treat various diseases. Over time, several studies have been conducted to investigate the functional properties of these products and their mode of action [1]. There is currently a wealth of evidence highlighting the potential of natural compounds in the prevention or treatment of aging [2], obesity and non-alcoholic fatty liver disease (NAFLD) [3, 4], neurodegenerative diseases [5] and numerous types of cancer [6, 7].

Polyphenols and flavonoids, such as curcumin, resveratrol, and phenolic acids, stand out among compounds with the strongest scientific evidence. These compounds have numerous pharmacological properties, including antioxidant, anti-inflammatory, antibacterial, immunomodulatory, anticancer, antiproliferative, antimutagenic, and antithrombotic activities [8–11].

Curcumin, derived from *Curcuma longa L*., a plant belonging to the Zingiberaceae family, stands out as one of the most extensively investigated polyphenols. In addition to curcumin (CUR) (about 75–80%), the plant contains approximately 50 other curcuminoids, including demethoxycurcumin (DMC) and bisdemethoxycurcumin (BDMC), which together comprise the primary bioactive constituents [12].

Despite its potent pharmacological activities, curcumin’s low bioavailability limits its effectiveness, prompting researchers to explore other curcuminoids, such as DMC and BDMC [13]. These compounds differ significantly from curcumin in both their chemical composition and biological activity, offering superior pharmacological effects [14]. BDMC, in particular, has been shown to possess more favorable properties than curcumin, in terms of bioavailability and bioaccessibility, leading to more potent therapeutic effects [15]. Moreover, these curcuminoids support curcumin’s biological activities and physical stability, improving its therapeutic potential. The combination of these three curcuminoids was found to be more effective since they act synergistically [12].

Among the therapeutic properties of curcuminoids, their cytotoxic activity has garnered significant attention [16]. Cancer remains a major global health challenge, with certain types, such as cutaneous melanoma, demonstrating resistance to conventional therapies and high recurrence rates, contributing to elevated mortality [17]. To solve this problem, several natural compounds, such as curcuminoids, and the modulation of alternative signaling pathways, such as purinergic signaling, have been studied as adjuvant strategies for treating the disease [18–20]. Cutaneous melanoma is characterized by its aggressive nature and ability to evade immune responses, often mediated by purinergic signaling pathways involving ectonucleotidases such as CD39 and CD73 [21]. These enzymes regulate extracellular ATP and adenosine levels, which play opposing roles in tumor immunity [22]. Targeting these pathways has emerged as a promising strategy for overcoming therapeutic resistance in melanoma.

**Fig. 1:**
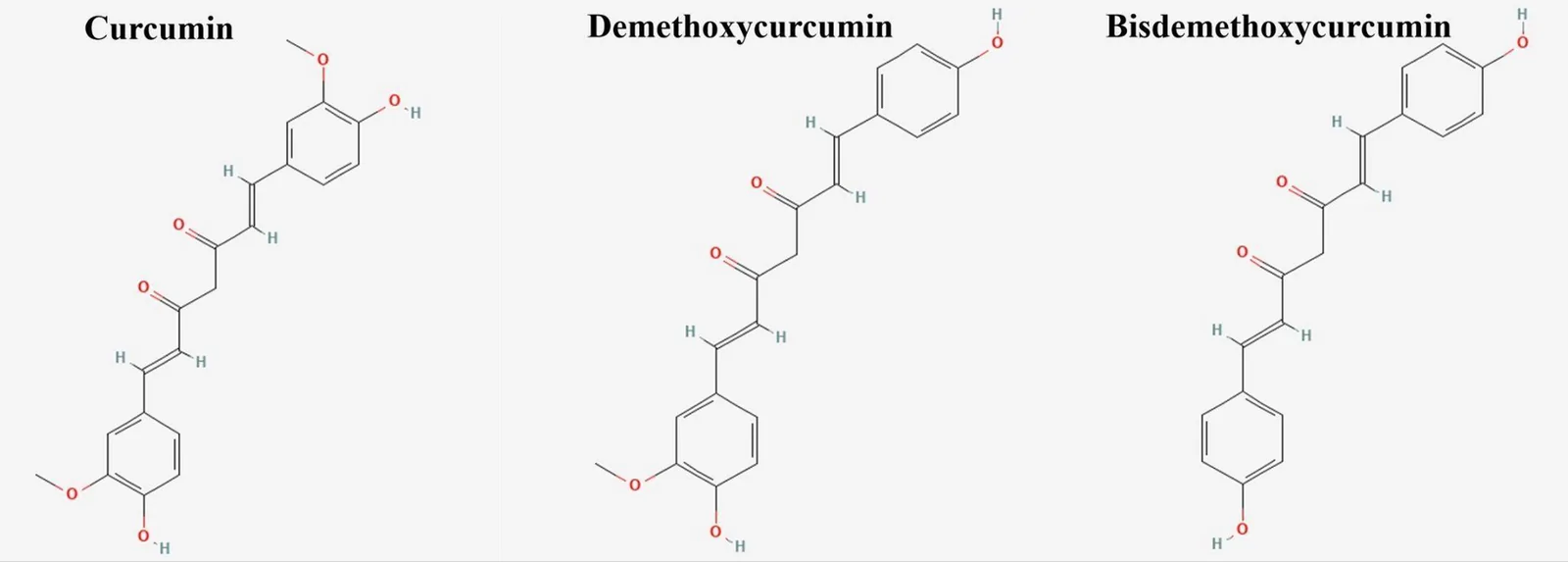
Chemical structures of curcumin, demethoxymethylcurcumin, and bisdemethoxymethylcurcumin. Source: PubChem

Considering the scarcity of results evaluating the antineoplastic activity of DMC and BDMC in metastatic cutaneous melanoma cell lines alone or in combination, the objective of the present study was to evaluate the cytotoxic and antiproliferative activity of the (Activated Curcumin C3 Complex^®^) (AC3^®^) (Sami-Sabinsa Group Limited) in two cutaneous melanoma cell lines, A375 and SK-MEL-28. Unlike regular turmeric extracts, it is a specialized formulation derived from Curcuma longa and enriched with DMC and BDMC to enhance physiological benefits. The chemical structures of Curcumin, Demethoxymethylcurcumin, and Bisdemethoxymethylcurcumin are shown in Fig. 1. Additionally, we sought to elucidate the key signaling pathways modulated by AC3^®^, with a focus on its effects on purinergic signaling, oxidative stress, and apoptotic mechanisms.

## Materials and methods

### Materials

Materials used in this study were high analytical grade purchased from Sigma-Aldrich (Sigma-Aldrich, St. Louis, MO, USA), Merck (Darmstadt, Germany), Thermo Fisher Scientific (Grand Island, NY, USA), Invitrogen Life Technologies (Carlsbad, CA, USA), and Sami-Sabinsa Group Limited (India). The cell lines A375 and SK-MEL-28 were purchased from the Cell Bank of Rio de Janeiro (BCRJ) (Brazil). Cell culture medium was purchased from Vitrocell™ (Brazil), and plates and flasks used for culture procedures were obtained from Kasvi™ (Brazil).

### Cell culture and exposure to AC3^®^

The A375 and SK-MEL-28 cell lines were cultured according to the recommendations of the Bank of Cell of Rio de Janeiro (BCRJ). For A375, Dulbecco’s Modified Eagle’s Medium (DMEM) containing 2mM of L-glutamine, 4500 mg/L glucose, and 10% of fetal bovine serum was used. For SK-MEL-28, we used Dulbecco’s Modified Eagle’s Medium (DMEM) supplemented with 2 mM L-glutamine, 1.0 g/L glucose, and 10% fetal bovine serum. The cells were maintained three times a week in a humidified, controlled atmosphere of 5% carbon dioxide (CO_2_) at 37 °C until reaching the desired confluence.

For assays performed in 96-well plates, a confluence of 1 × 10^4^ was used, while for the cell migration and molecular biology assays, 1 × 10^6^ cells/well were used. AC3^®^ was dissolved in 0.2% dimethyl sulfoxide (DMSO); the concentrations used (0.78 µg/mL, 1.56 µg/mL, 3.12 µg/mL, 6.25 µg/mL) were defined through a previous study [32]. Cells in the negative control (CT) group were treated with culture medium only. After treatment, all treatment groups were incubated for 24 h.

AC3^®^ (Activated Curcumin C3 Complex^®^), an orange-yellow powder, is a unique formulation of bisdemethoxycurcumin (BDMC) enriched Curcuma longa extract, obtained through solvent extraction, and is standardized to contain a minimum of 85–95% w/w of total curcuminoids by HPLC. In terms of purity, AC3^®^ contains 30–40% percent BDMC, 15–25% demethoxycurcumin, and 40–50% curcumin.

### Cell viability by MTT assay

To assess cell viability, the 3-(4,5-dimethyl-2-thiazolyl)-2,5-diphenyl-2 H-tetrazolium bromide (MTT) assay was used as previously described by Mosmann [23]. After the treatment exposure time, the treatment was removed, the cells were washed with phosphate-buffered saline (PBS) (0.1 M, pH 7.4), and 100 µL of MTT dissolved in PBS was added. The cells were incubated at 37 °C for 2 h, during which time the viable cells reduced the MTT to formazan crystals, which were subsequently dissolved in DMSO and read at 570 nm in a microplate reader (Varioskan™ LUX multimode microplate reader (Thermo Fisher Scientific, Waltham, MA, USA).

### Cell viability by fluorescence microscopy assay

Cell viability was also measured using the fluorophore acridine orange (AO), which is taken up only by viable cells and stains both double-stranded (ds) and single-stranded (ss) nucleic acids. Consequently, viable cells emit green fluorescence, which is read under a fluorescence microscope (Nikon^®^ Eclipse TS2-FL) (480–490 nm) in triplicate at 200× magnification and adjusted for brightness and contrast linearly by Imagej^®^ software [24].

### Measurement of mitochondrial transmembrane potential (ΔΨm)

The mitochondrial transmembrane potential was measured using tetramethylrhodamine ethyl ester (TMRE), which accumulates in active mitochondria and emits red fluorescence proportional to the mitochondrial transmembrane potential when excited at 550 nm. All samples were read in triplicate under a fluorescence microscope (Nikon^®^ Eclipse TS2-FL) at 200× magnification and adjusted for brightness and contrast linearly using Imagej^®^ software [25].

### Wound-healing migration assay

The cell migration assay was performed as described by Justus et al. [26], with adaptations. Briefly, A375 and SK-MEL-28 cells were seeded at 1 × 10^6^ cells/well in 6-well plates and grown to 100% confluence. A linear scratch was then made across the cell monolayer using a sterile 200 µL pipette tip. Detached cells and debris were removed by gently washing the wells twice with sterile PBS. An initial image (T = 0 h) of the scratch was acquired by inverted optical microscopy (Nikon^®^ Eclipse TS2-FL, Nikon Corporation, Tokyo, Japan) at 4× magnification. Cells were then treated with AC3^®^ at the indicated concentrations or with vehicle (0.2% DMSO) for 24 h. After treatment, the medium was discarded, the cells were gently washed once with PBS, and a final image (T = 24 h) of the same field was acquired under identical conditions. Wound closure was quantified using ImageJ^®^ v1.53t (National Institutes of Health, Bethesda, MD, USA) and expressed as percentage closure relative to T = 0 h, normalized to the negative control (set as 100%). Three independent biological replicates were performed in triplicate technical replicates.

### Detection of reactive oxygen species (ROS)

ROS levels were checked by a fluorescent assay using 2,7-dichlorodihydrofluorescein diacetate (H_2_DCF-DA), as previously described by Manica et al. [27]. The reading was performed on a fluorescence plate reader (Varioskan™ LUX multimode microplate reader (Thermo Fisher Scientific, Waltham, MA, USA)) at Ex./Em. = 488/525 nm. Three independent biological replicates (*n* = 3) were performed, each in quadruplicate, and results were expressed as a percentage (%) of relative fluorescence compared to the control.

### Gene expression

To extract total RNA from cells, TRIzol^®^ reagent (Invitrogen) was used according to the manufacturer’s instructions. Subsequently, RNA quantification and purity verification were performed using the 260/280 nm absorbance ratio with a µDrop™ spectrophotometer Varioskan™ LUX multimode microplate reader (Thermo Fisher Scientific, Waltham, MA, USA). RNA was treated with DNase (Thermo Scientific, USA) following the manufacturer’s recommendations. cDNA was prepared according to the instructions of the cDNA High-Capacity cDNA Reverse Transcription Kit (Thermo Scientific, USA). To evaluate gene expression, each reaction consisted of 6 µL of cDNA sample, 10 µL of PowerUp™ SYBR™ Green Master Mix for qPCR (Thermo Scientific, USA), 2 µL of primer F (500 nM), and 2 µL of primer R (500 nM) for a final volume of 20 µL. Amplification was performed on the QuantStudio™ 7 Pro Real-Time PCR System (Thermo Scientific, USA). Melting curve analysis was performed to verify product identity. Tests were conducted in quadruplicate, and GAPDH was used as a housekeeping gene. A calibrator was used, and relative gene expression was determined using the comparative 2 − ΔΔCq method, equivalent to the 2 − ΔΔ^Ct^ method described by Livak and Schmittgen (2001), with the negative control set to 1.0 as reference [28]. The forward and reverse (5’-3’) oligo sequences used for each gene are described in Table 1.

**Table 1:** Primer sequences for RT-qPCR

Gene	GenBank ID	Forward (5’-3’)	Reverse (5’-3’)	Amplicon (bp)
GAPDH	NM_002046	CTCCTCACAGTTGCCATGTA	GTTGAGCACAGGGTACTTTATTG	143
CASP-8	NM_001228	AGGAGCTGCTCTTCCGAATT	CCCTGCCTGGTGTCTGAAGT	115
CASP-3	NM_004346	TTTGAGCCTGAGCAGAGACATG	TACCAGTGCGTATGGAGAAATGG	147
TNF-α	NM_000594	CAGGCAGTCAGATCATCTTC	GCTTGAGGGTTTGCTACAAC	164
IL-6	NM_000600	TCATCCCATAGCCCAGAGCA	CTGGCATTTGTGGTTGGGTC	190
NLRP3	NM_004895	AACATGCCCAAGGAGGAAGA	GGCTGTTCACCAATCCATGA	152
CD39 (ENTPD1)	NM_001098175	GCCCTGGTCTTCAGTGTATTAG	CTGGCATAACCTACCTACTCTTTC	138
CD73 (NT5E)	NM_002526	GTGCCTTTGATGAGTCAGGTAG	TTCCTTTCTCTCGTGTCCTTTG	171

Glyceraldehyde-3-phosphate dehydrogenase (GAPDH), Caspase-8 (CASP-8). Caspase-3 (CASP-3), Tumor Necrosis Factor Alpha (TNF-α), Interleukin 6 (IL-6), NLR Family Pyrin Domain Containing 3 (NLRP3), Ectonucleoside triphosphate diphosphohydrolase 1 (CD39), Ecto-5′‐nucleotidase (CD73), Adenosine deaminase (ADA).

### Assessment of CD39 and CD73 enzymatic activities

The enzymatic activities of CD39 and CD73 were performed according to Pilla et al. [29] and Lunkes et al. [30] with adaptations. The reactions were performed in quadruplicate, prepared as described [31], and subsequently read at 630 nm using the Varioskan™ LUX multimode microplate reader (Thermo Fisher Scientific, Waltham, MA, USA).

A standard curve with KH_2_PO_4_ was used to calculate the enzymatic activity, and the results were expressed as nmol of Pi/min/mg protein.

### Protein determination

The Bradford method [32] was employed for protein determination, using bovine serum albumin as the standard. The protein samples were adjusted to the concentration required for each assay in mg/mL.

### Statistical analysis

All experiments were performed in three independent biological replicates (*n* = 3), each with the technical replicates indicated for each assay (MTT and ROS: quadruplicate technical replicates; fluorescence microscopy: triplicate; wound-healing assay: triplicate; RT-qPCR: technical quadruplicates; CD39/CD73 enzymatic activity: quadruplicate). Data are presented as mean ± standard deviation (SD). Normality of data distribution was assessed using the Shapiro-Wilk test. When the data met the assumption of normality, group comparisons were performed using one-way analysis of variance (ANOVA), followed by Dunnett’s *post hoc* test, which corrects for multiple comparisons against the negative control. When the data did not meet the assumption of normality, the Kruskal-Wallis test, followed by Dunn’s post hoc test, was applied. Differences with *P* < 0.05 were considered statistically significant: * (*P* < 0.05), ** (*P* < 0.01), *** (*P* < 0.001), **** (*P* < 0.0001). For RT-qPCR analyses, fold-change values are reported alongside P-values in the Results section. All statistical analyses were performed using GraphPad Prism v9.0 (GraphPad Software Inc., San Diego, CA, USA).

## Results

### AC3^®^ decreased the viability and migration of melanoma cells

**Fig. 2:**
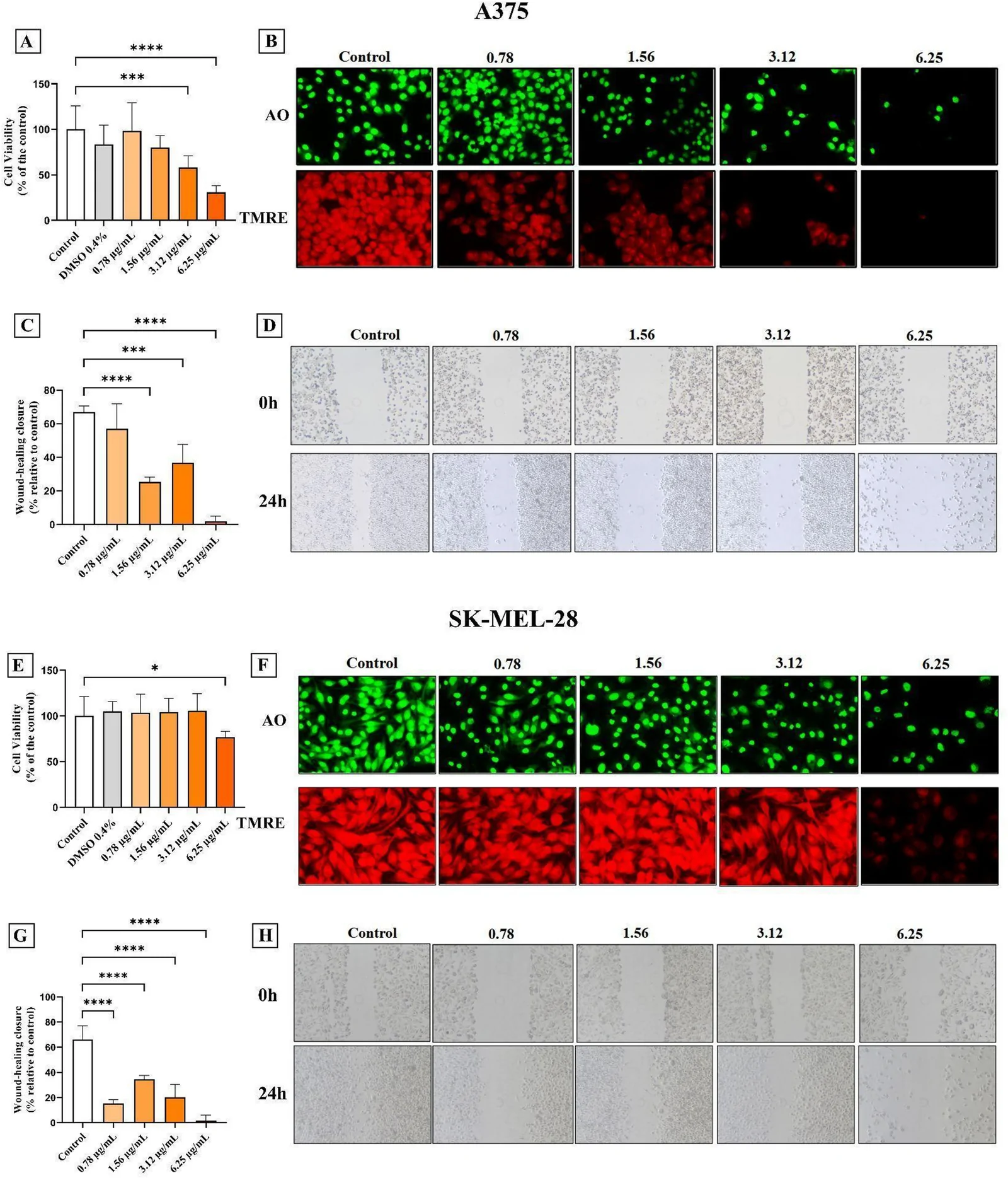
Cytotoxic and migratory activity of AC3^®^ in CM A375 and SK-MEL-28 cells. In the A375 cell line, the MTT assay indicated that AC3^®^ concentrations of 3.12 µg/mL and 6.25 µg/mL reduced cell viability after 24 h of treatment (**A**); similar results were observed by fluorescence microscopy (**B**). In the cell migration assay, we observed reduced cell migration at concentrations of 1.56 µg/mL, 3.12 µg/mL, and 6.25 µg/mL (**C**, **D**). In the SK-MEL-28 cell line, we found that treatment with 6.25 µg/mL of AC3 decreased cell viability (**E**, **F**). Furthermore, all treatment concentrations effectively reduced CM cell migration (**G**, **H**). Data are presented as mean ± SD. Statistical analysis: ANOVA. Values with *P* < 0.05 were considered statistically significant. *(*P* < 0.05) **(*P* < 0.01) ***(*P* < 0.001) ****(*P* < 0.0001)

Figure 2 presents the cytotoxic effect of AC3^®^ on the SK-MEL-28 and A375 cells. AC3^®^ was significantly cytotoxic and inhibited the migration of A375 and SK-MEL-28 cells. In the A375 cell line, a significant reduction in cell viability was observed at concentrations of 3.12 µg/mL (*P* = 0.0009) and 6.25 µg/mL (*P* < 0.0001) (Fig. 2A).

The decrease in cell viability was further supported by fluorescence microscopy, in which nuclei were stained with the fluorescent dye acridine orange (AO, green) and mitochondria with Tetramethylrhodamine Methyl Ester (TMRE, red). A decrease in fluorescence intensity indicated cytotoxic effect. Moreover, the wound-healing assay demonstrated an inhibition of A375 cell migration following treatment with AC3^®^, with significant effects observed at concentrations of 1.56 µg/mL (*P* < 0.0001), 3.12 µg/mL (*P* = 0.0003), and 6.25 µg/mL (*P* < 0.0001) (Fig. 2C-D).

Similarly, in the SK-MEL-28 cutaneous melanoma cell line, AC3^®^ demonstrated a significant reduction in cell proliferation at the highest concentration tested (*P* = 0.0304) (Fig. 2E-F). Furthermore, the wound-healing assay indicated an inhibition of cell migration, as evidenced by a significant reduction in wound closure across all tested concentrations (*P* < 0.0001) (Fig. 2G-H).

### AC3^®^ decreases ROS levels and modulates caspase expression in CM A375 and SK-MEL-28 cells

**Fig. 3:**
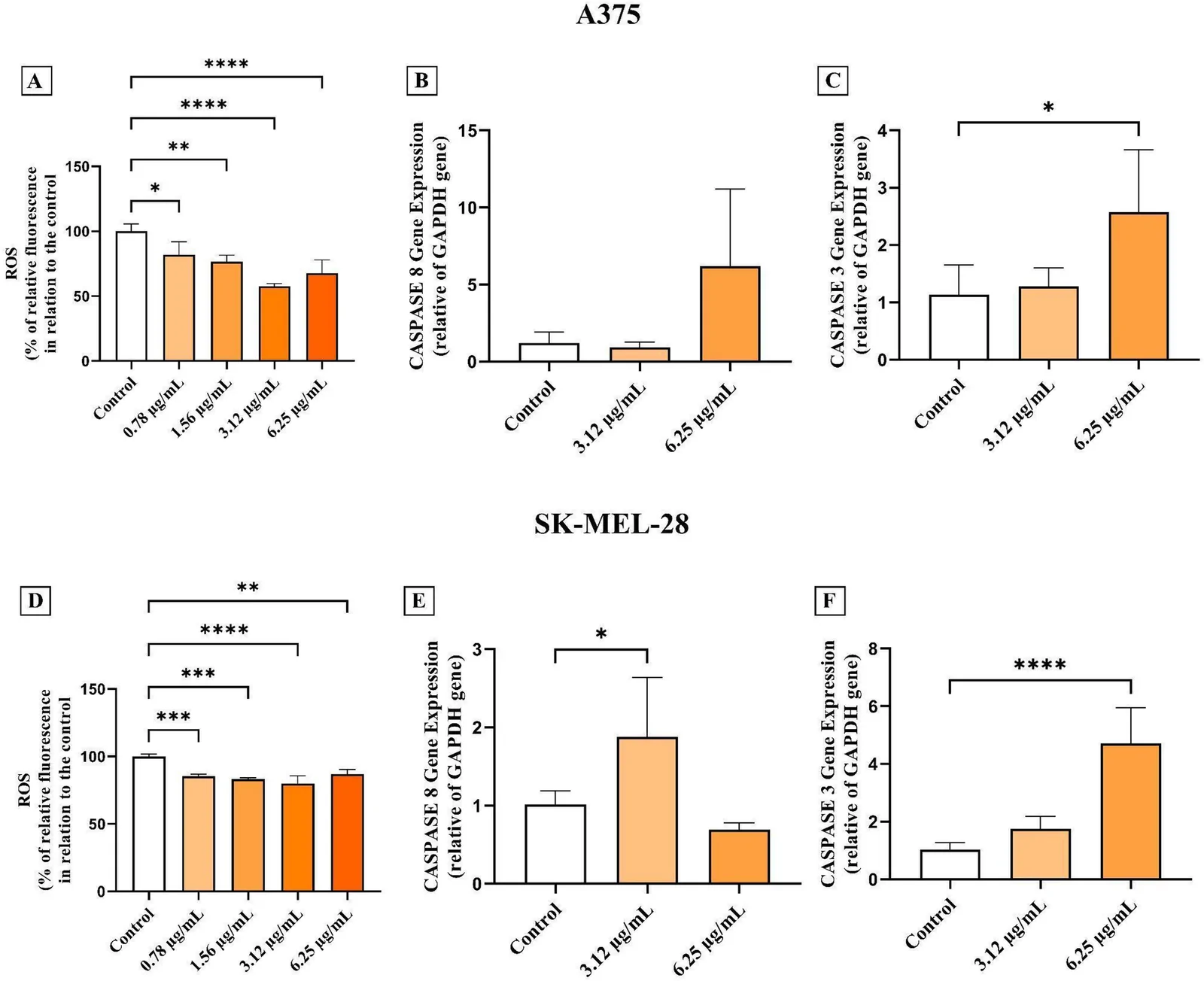
AC3^®^ modulates ROS levels and caspase expression in CM A375 and SK-MEL-28 cells. Treatment with all AC3^®^ concentrations reduced ROS levels in A375 (**A**) and SK-MEL-28 (**D**) cell lines. We did not observe a significant change in Caspase-8 gene expression levels in A375 (**B**) cells, whereas in SK-MEL-28 cells, treatment with 3.12 µg/mL AC3^®^ increased gene expression (**E**). Caspase-3 gene expression significantly increased after treatment with 6.25 µg/mL of AC3 in A375 (**C**) and SK-MEL-28 (**F**) cells. Data are presented as mean ± SD. Statistical analysis: ANOVA. Values with *P* < 0.05 were considered statistically significant. *(*P* < 0.05) **(*P* < 0.01) ***(*P* < 0.001) ****(*P* < 0.0001)

Following a 24-h treatment with AC3^®^, a significant reduction in reactive oxygen species (ROS) levels was observed across all tested concentrations in both A375 (Fig. 3A) and SK-MEL-28 (Fig. 3D) melanoma cell lines. To further elucidate the AC3^®^ mechanism of action, the gene expression of caspases 8 and 3 was evaluated. In SK-MEL-28 cells, treatment with AC3^®^ at 3.12 µg/mL resulted in a significant upregulation of caspase-8 gene expression (*P* = 0.0435) (Fig. 3E). In contrast, no significant alterations in caspase-8 expression were detected in A375 cells when compared to the control (Fig. 3B). Regarding caspase 3, we found a substantial increase in caspase-3 gene expression after treatment with 6.25 µg/mL of the compound in the A375 (*P* = 0.0150) (Fig. 3C) and SK-MEL-28 (*P* < 0.0001) (Fig. 3F) cell lines.

### AC3^®^ modulates the gene expression of components of the inflammatory cascade

**Fig. 4:**
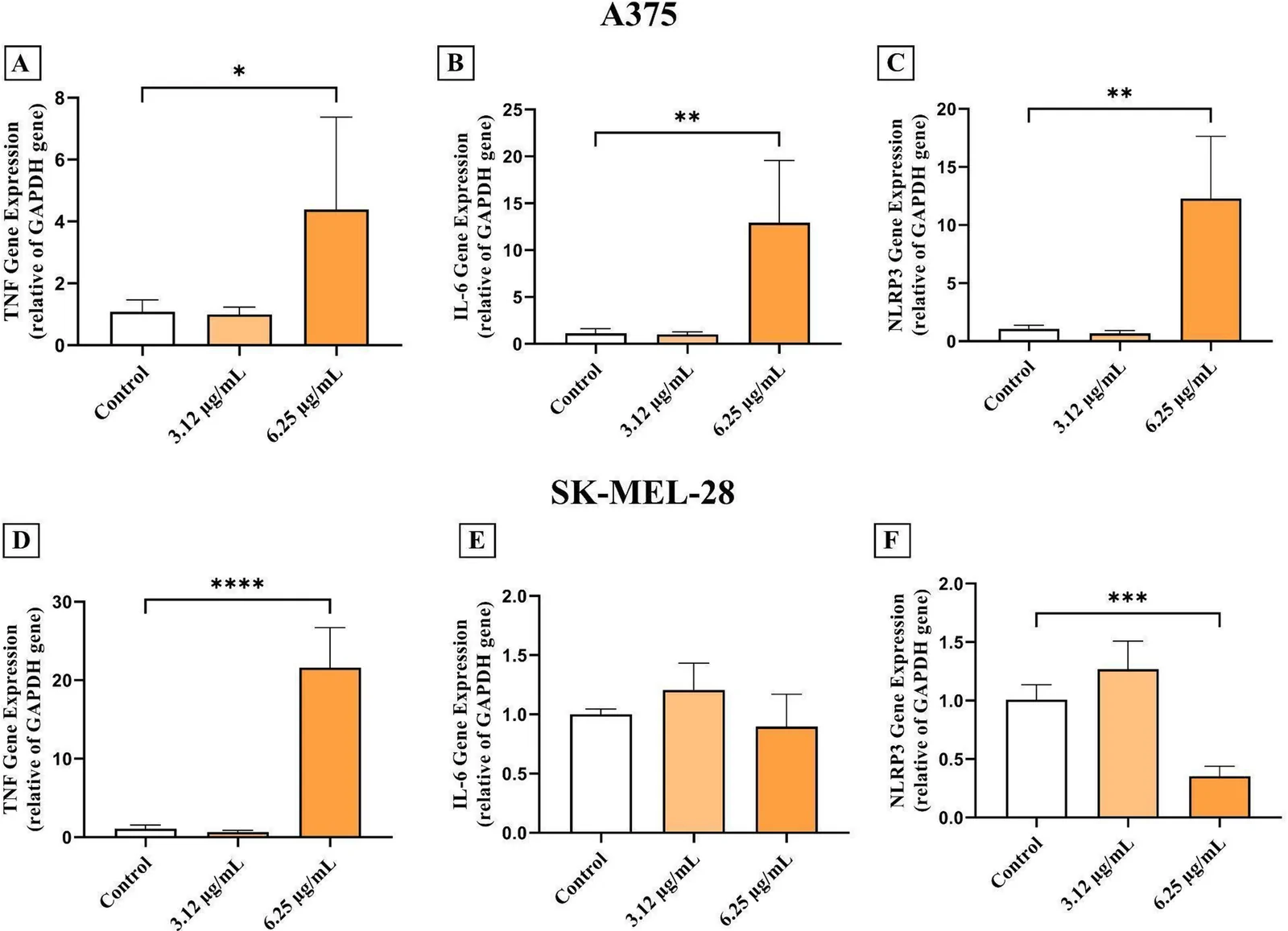
AC3^®^ modulates the levels of pro-inflammatory markers in CM A375 and SK-MEL-28 cell lines. In A375 cells, treatment with 6.25 µg/mL of AC3^®^ increased the gene expression of TNF (**A**), IL-6 (**B**), and NLRP3 (**C**). In SK-MEL-28 cells, we observed similar results in TNF levels (**D**). In contrast, IL-6 levels (E) did not differ significantly, whereas NLRP3 gene expression decreased after 24 h of treatment with 6.25 µg/mL AC3^®^ (**F**). Data are presented as mean ± SD. Statistical analysis: ANOVA. Values with *P* < 0.05 were considered statistically significant. *(*P* < 0.05) **(*P* < 0.01) ***(*P* < 0.001) ****(*P* < 0.0001)

We used RT-qPCR to evaluate TNF-α, IL-6, and NLRP3 gene expression, as shown in Fig. 4. After 24 h of treatment with 6.25 µg/mL of AC3^®^, there was an increase in the gene expression of TNF-α (*P* = 0.0446), IL-6 (*P* = 0.0035), and NLRP3 (*P* = 0.0012) in the A375 cell line (Fig. 4A-C). In the SK-MEL-28 cell line, an increase in the gene expression of TNF-α (*P* < 0.0001) was observed at the highest treatment concentration (Fig. 4D). In contrast, in this same cell line and treatment concentration, a reduction in the levels of NLRP3 was observed (*P* = 0.0006) (Fig. 4F).

### AC3^®^ modulates gene expression of CD39 and CD73

**Fig. 5:**
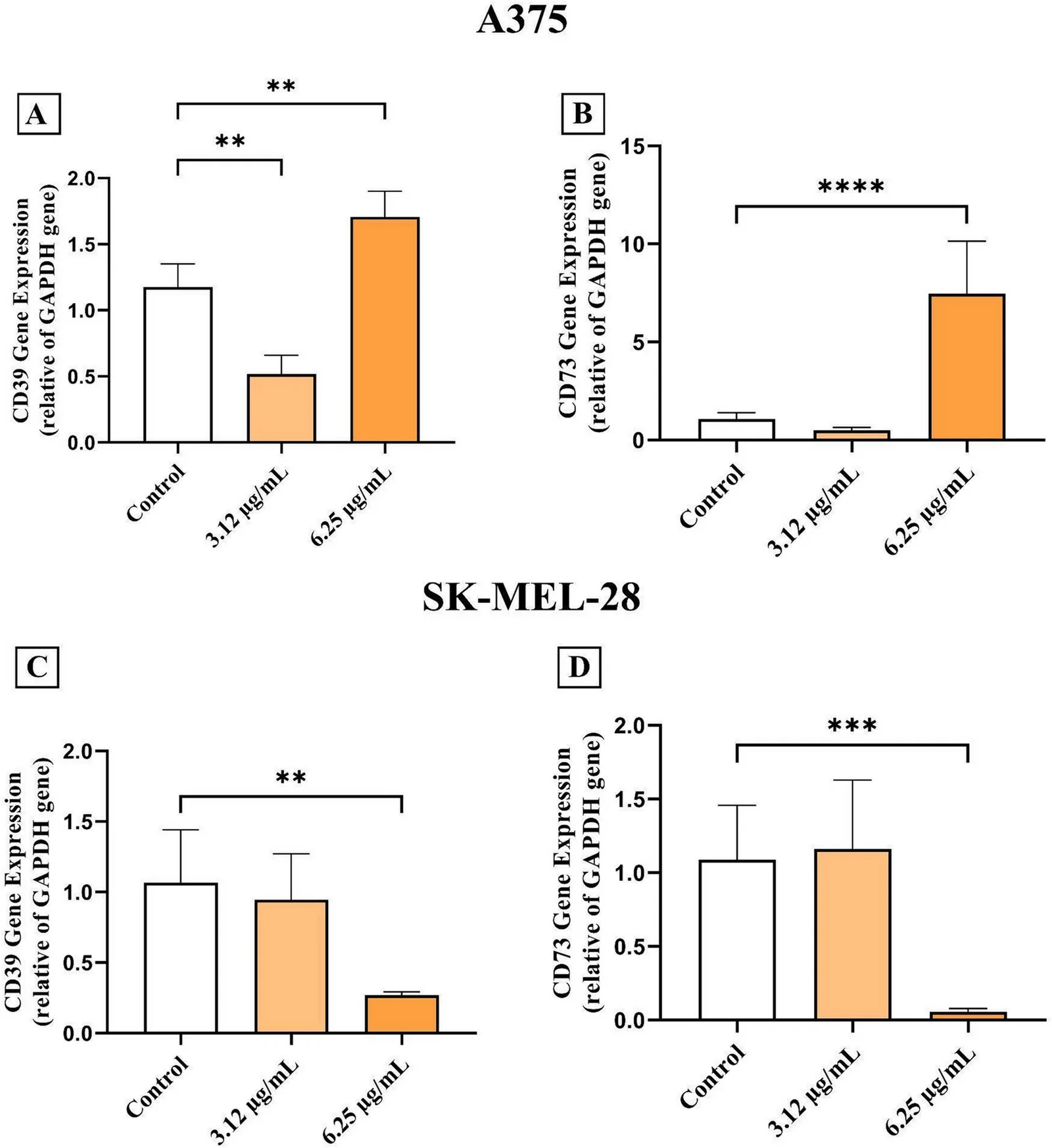
AC3® modulates the levels of pro-inflammatory markers in CM A375 and SK-MEL-28 cell lines. CD39 gene expression levels increased after AC3® (A) treatment in A375 cells. The same was observed for CD73 levels after treatment with 6.25 µg/mL of the compound (B). The highest concentration tested in the SK-MEL-28 cell line decreased CD39 (C) and CD73 (D) gene expression. Data are presented as mean ± SD. Statistical analysis: ANOVA. Values with P < 0.05 were considered statistically significant. *(P < 0.05) **(P < 0.01) ***(P < 0.001) ****(P < 0.0001)

We evaluated the gene expression of ectonucleotidases after 24 h of treatment with AC3^®^ in A375 and SK-MEL-28 cells, as shown in Fig. 5 (A-D). In A375 cells, the treatment concentration of 3.12 µg/mL (*P* = 0.0011) decreased CD39 expression. In comparison, at the concentration of 6.25 µg/mL (*P* = 0.0078), there was an increase in CD39 gene expression (Fig. 5A). Regarding CD73, the concentration of 6.25 µg/mL increased gene expression in A375 cells (*P* < 0.0001) (Fig. 5B). Differently, for SK-MEL-28 cells, the treatment was able to decrease CD39 gene expression at the concentration of 6.25 µg/mL (*P* = 0.0016) (Fig. 5C). Similarly, treatment with 6.25 µg/mL of AC3^®^ decreased CD73 gene expression, compared to the control (*P* = 0.0009) (Fig. 5D).

### AC3^®^ modulates enzymatic activity of CD39 and CD73

**Fig. 6:**
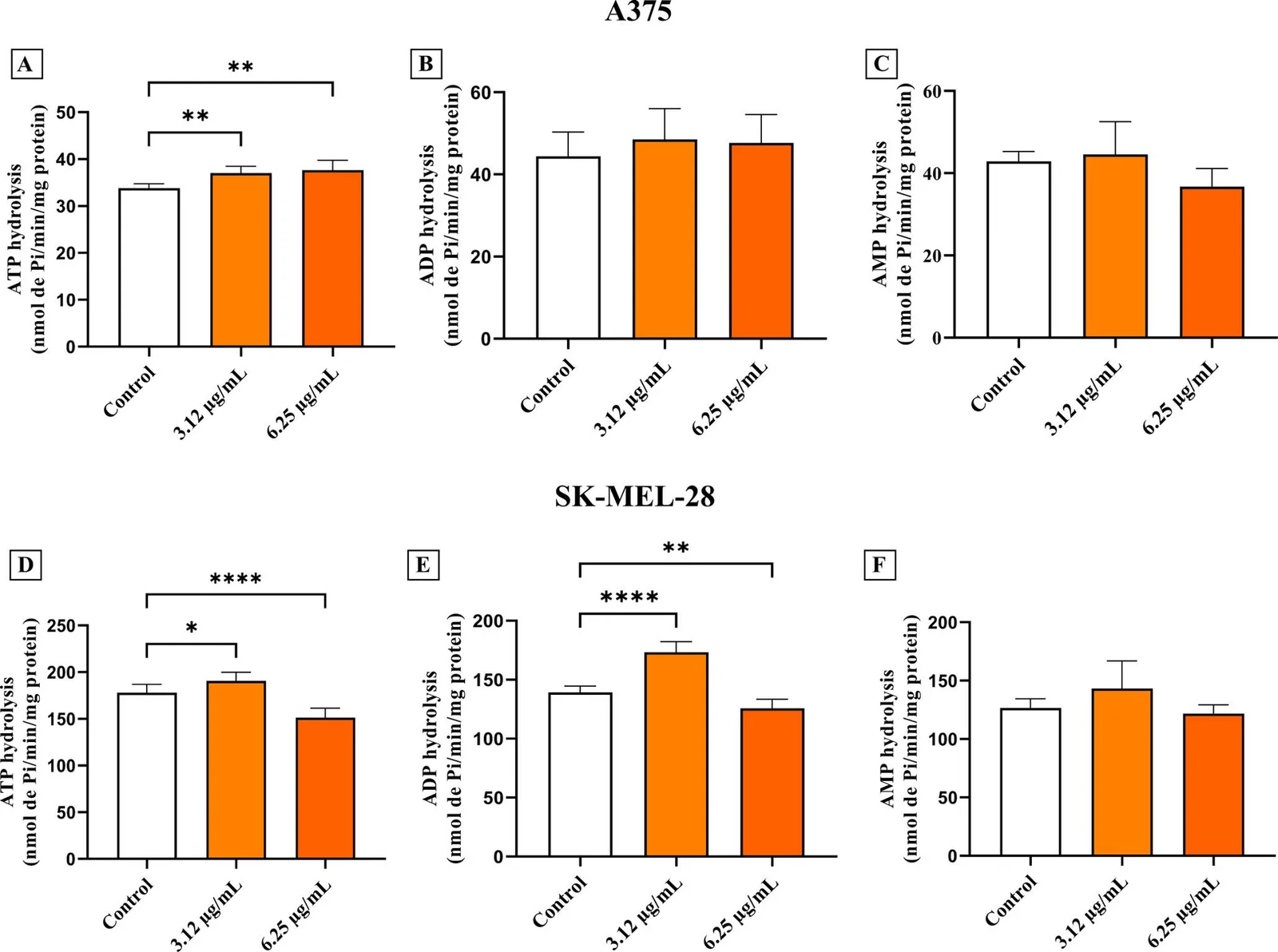
AC3® modulates the enzymatic activity of CD39 and CD73 in CM A375 and SK-MEL-28 cell lines. Regarding enzymatic activity, we observed increased ATP hydrolysis after AC3® treatment in the A375 cell line (A). In contrast, in SK-MEL-28 cells, we observed an increase in ATP hydrolysis at a concentration of 3.12 µg/mL, followed by a decrease at 6.25 µg/mL (D). The same occurred in the levels of ADP hydrolysis (E). We did not observe significant differences in ADP (B) and AMP (C) hydrolysis in the A375 cell line, or in AMP hydrolysis (F) in SK-MEL-28 cells. Data are presented as mean ± SD. Statistical analysis: ANOVA. Values with P < 0.05 were considered statistically significant. *(P < 0.05) **(P < 0.01) ***(P < 0.001) ****(P < 0.0001)

Figure 5 shows the results of the enzymatic activity of CD39 and CD73 after 24 h of treatment with AC3^®^ (Fig. 6A-F). In the A375 cell line, an increase in ATP hydrolysis was observed after treatment with 3.12 µg/mL (*P* = 0.0059) and 6.25 µg/mL (*P* = 0.0015) (Fig. 6A). No significant difference was observed in the levels of ADP (Fig. 6B) and AMP (Fig. 6C) hydrolysis in this cell line after treatment with the compound.

In SK-MEL-28 cells, after 24 h of treatment with AC3^®^, we observed an increase in ATP hydrolysis at the concentration of 3.12 µg/mL (*P* = 0.0251), followed by a decrease at the concentration of 6.25 µg/mL (*P* < 0.0001) (Fig. 5D). Similar results were obtained in AMP hydrolysis, where an increase in CD39 activity occurred at a concentration of 3.12 µg/mL (*P* < 0.0001), followed by a decrease at a concentration of 6.25 µg/mL (*P* = 0.0061) (Fig. 5E). Similarly to the A375 cell line, CD73 activity did not show significant differences after treatment with AC3^®^.

## Discussion

Curcuminoids have been used for many years as a spice. Among them, the most studied curcuminoid is CUR, which has shown cytotoxic effects in several types of cancer, such as breast [33], ovarian [34], esophagus [35], colorectal [36] and pancreatic [37]. In melanoma cells lines, it was proved that curcumin induces apoptosis by activating cell death pathways involving caspases 3, 7, 8, and 9, and AKT/mTOR. It also can decrease the expression of factors associated with angiogenesis and invasion, such as Vascular Endothelial Growth Factor (VEGF), Matrix Metalloproteinase-2 (MMP- 2), Matrix Metalloproteinase-9 (MMP-9), and Cyclooxygenase (COX), which are predictive of metastasis [27–40]. The others curcuminoids, such as demethoxycurcumin (DMC) and bisdemethoxycurcumin (BDMC), also have cytotoxic activity and have been shown to act synergistically with curcumin [16, 41–43]. Considering the previous cytotoxic activity of available commercial curcuminoids, we verified the cytotoxic properties of a mixture of phytonutrients that expounds the benefits of Bisdemethoxycurcumin (BDMC), as the major ingredient, along with Demethoxycurcumin (DMC) and Curcumin standardized in AC3^®^ in two cutaneous melanoma cell lines. The AC3^®^ used in this study was obtained by solvent extraction of Curcuma Longa enriched in bisdemethoxycurcumin (BDMC) and is standardized by HPLC to contain at least 85% w/w total curcuminoids. While the crude extract of C. longa includes 2.2% to 6.5% BDMC, the AC3^®^ sample contains between 30% and 35% of this compound. Furthermore, AC3^®^ has already been subjected to a battery of in vivo toxicological assays—acute, subacute, subchronic, reproductive/developmental, and genotoxicity—and has shown no genotoxic effects in rodents [44].

Moreover, in our study, we selected two cutaneous melanoma cell lines, A375 and SK-MEL-28. These cell lines are widely studied and present different levels of aggressiveness. A comparative study of cutaneous melanoma cell lines using an aggressiveness scale (Melanoma AGgressiveness Score: MAGS) showed that the A375 cell line was classified as the most aggressive, and the SK-MEL-28 cell line was the least aggressive [45].

In the present study, AC3^®^ decreased cell proliferation and migration in both melanoma cell lines. According to Huang et al. [16], the three curcuminoid constituents (CUR, DMC, and BDMC) act synergistically when combined. The present results corroborate previous reports showing that curcuminoids exert dose-dependent inhibitory effects on proliferation and migration of cutaneous melanoma cell lines, including A375 and SK-MEL-28 [41, 46, 47]. Similarly, curcumin-loaded nanoparticles have demonstrated antiproliferative and anti-migratory effects, along with a marked reduction in cell viability in breast cancer models [48]. In the present work, a dose-dependent decrease in cell viability was observed, with statistically significant cytotoxicity at the higher concentrations tested (3.12 and 6.25 µg/mL for A375; 6.25 µg/mL for SK-MEL-28; Fig. 2).

In addition to reducing cell viability, previous studies have found that curcuminoids increase ROS levels, thereby damaging DNA and inducing apoptosis [49, 50]. In contrast to the results found here, we found that when in association, these compounds decrease ROS levels in A375 and SK-MEL-28 cells and increase caspases-8 and − 3 expression independent of ROS levels (Fig. 3). Sandur et al. [12] found similar results, in which, regardless of ROS levels, curcuminoids induced cell death via nuclear factor-κB (NF-kB) and TNF, that is, apoptosis through the extrinsic pathway. Our data suggest that AC3^®^ exerts its cytotoxic effects, at least in part, by reducing oxidative stress and modulating the apoptotic machinery through regulation of caspase gene expression in melanoma cells.

It should be noted that the relationship between curcuminoids, ROS, and apoptosis is biphasic and context-dependent. Whereas several studies report curcumin-induced apoptosis through ROS-dependent mechanisms in melanoma [49, 50], a parallel body of evidence describes ROS-independent or even ROS-suppressive cytotoxicity, particularly when curcuminoid mixtures or BDMC-enriched extracts are used. Sandur et al. [12] demonstrated that the curcumin/DMC/BDMC mixture differentially regulates anti-inflammatory and antiproliferative responses via a ROS-independent mechanism involving NF-κB and TNF signaling, a finding that parallels our findings. Hu et al. described ROS-independent DNA damage and G2/M arrest induced by curcumin in BCPAP thyroid carcinoma cells [51]. The decrease in ROS observed at concentrations that simultaneously upregulate caspase-3/-8 and TNF-α transcripts in the present study is therefore consistent with engagement of a non-ROS-mediated apoptotic program likely driven by death receptor signaling and NF-κB-dependent transcription. The apparent paradox between decreased ROS and increased apoptotic markers warrants direct mechanistic dissection. Pharmacological strategies, including ROS scavengers (e.g., N-acetylcysteine, Trolox) or pro-oxidants, would be needed to formally establish whether the observed ROS reduction is causally linked to or independent of the pro-apoptotic gene expression changes; this is acknowledged as a limitation of the present study.

Furthermore, the reduction in ROS is directly linked to metalloproteinases, proteins closely associated with cell migratory potential, which may explain an antimigratory effect even at lower compound concentrations [10].

An increase in TNF-α gene expression was observed after treatment with AC3^®^ in the A375 and SK-MEL-28 cells (Fig. 4). In these cases, apoptosis is mediated via TNF and carried out by the TNF receptor 1 (DR1/TNFR1), in which interaction occurs between TNF and TNFR1 leading to the recruitment of the TNFR-associated death domain (TRADD) protein, through its DD [52]. Subsequently, interaction occurs with the Fas-associated death domain (FADD), which recruits pro-caspase-8 and cleaves it, generating active caspase-8, which in turn activates caspase-3, an effector of cell death [53]. In A375 cells, Tyciakova et al. [54] demonstrated that TNF overexpression is correlated with an increase in interleukin-6 (IL-6) and the pro-apoptotic tumor necrosis factor ligand gene (TRAIL) mediated by TNF/TNFR1 signaling. Similar results were observed in our study, in which we observed an increase in the inflammatory cascade and overexpression of TNF, IL-6, and NLRP3 in the cell line A375.

Importantly, the response of melanoma cells to TNF-α is often blunted by elevated expression of cellular FLICE-like inhibitory protein (c-FLIP), which competes with procaspase-8 for binding to FADD at the death-inducing signaling complex (DISC), thereby inhibiting extrinsic apoptosis [55, 56]. In BRAF V600E cutaneous melanoma, TNF-α activates NF-κB and upregulates c-FLIP, conferring resistance to RAF inhibitor- and TNF-induced apoptosis [57, 58]. This c-FLIP–mediated resistance may partly explain why the caspase-8 transcript was not significantly upregulated in the more aggressive A375 cell line in the present study, despite robust TNF-α induction. Notably, curcuminoids have been reported to downregulate NF-κB and c-FLIP-related signaling in other tumor models [12], suggesting that AC3^®^ may potentially overcome this resistance — a hypothesis warranting further validation through c-FLIP protein assessment, NF-κB activity assays, and pharmacological blockade experiments. Furthermore, the caspase-3 and caspase-8 findings reported here reflect only transcriptional modulation; protein-level activation (cleaved caspases) and enzymatic activity assays will be required in future work to confirm functional engagement of the apoptotic machinery.

Another essential component of the inflammatory cascade is the NLRP3 inflammasome, considered a double-edged sword in several types of cancer, capable of exerting both protective antitumorigenic and pro-tumorigenic effects [59]. In CM, the NLRP3 inflammasome is highly expressed, promoting tumor progression by activating and cleaving caspases and IL-1β, attenuating the immune response, and consequently suppressing anti-melanoma T cell responses [53, 60, 61]. In our previous study on the SK-MEL-28 cell line treated with CUR [20], we observed that the extract could decrease NLRP3 expression and, consequently, increase the efficacy of anti-melanoma therapies, particularly those targeting the anti-programmed cell death protein 1 (anti-PD-1) pathway. Contrary to what was found in A375 cells, in the SK-MEL-28 cell line, we observed a decrease in the gene expression of the NLRP3 inflammasome, supporting the hypothesis of the previous study that in this type of melanoma, treatment with CUR or a mix of curcuminoids CUR, DMC and BDMC, in this case, AC3^®^ can improve the response to therapies, acting mainly in the inhibition of tumor suppression, that is, positively regulating the immune response [62].

Another important therapeutic target for the treatment of CM, one capable of modulating the immune response, is purinergic signaling, particularly the extracellular purinergic mediators adenosine triphosphate (ATP) and adenosine (Ado) [63, 64]. ATP present in the extracellular environment can be released in different ways. One of them is cell lysis, a process that is directly related to apoptosis or by non-lytic mechanisms such as exocytosis of ATP-containing vesicles via nucleotide-permeable channels (connexin/pannexin hemichannels, maxi-anionic channels, volume-regulated anion channels, and P2 × 7 receptor channels), through transport vesicles that deliver proteins to the cell membrane or through lysosomes [65, 66]. Ado is generated by the dephosphorylation of ATP by the ectonucleotidases ectonucleoside triphosphate diphosphohydrolase 1 (CD39) and ecto-5’-nucleotidase (CD73), the former converting ATP into adenosine monophosphate (AMP), and the latter converting the AMP resulting from CD39 activity into Ado [76]. High levels of ATP in the tumor microenvironment act as a Danger-Associated Molecular Pattern (DAMP), inducing the immune response. Otherwise, Ado inhibits the immune response, causing immunosuppression [67, 68]. Antitumor immunity induced by Ado is a significant challenge in the treatment of CM, since newly available therapies that inhibit immune checkpoints (ICIs) and offer more favorable prognoses depend on the negative regulation of the CD39/CD73/Ado axis [69, 70].

Patients with CM have higher levels of CD39 and CD73 expression, which are associated with resistance to therapies and worse prognosis [71, 72]. In this study, we found that treatment with 6.25 µg/mL of the AC3^®^ complex was able to decrease the gene expression of the ectonucleotidases CD39 and CD73in the SK-MEL-28 cell line, considered the least aggressive (Fig. 4). Furthermore, in this same lineage we observed a decrease in the activity of the CD39 enzyme (Fig. 5). In contrast, in the A375 cell line, there was an increase in the expression of the CD39 and CD73 enzymes and in the enzymatic activity of CD39 (Fig. 4–5), demonstrating that the extract can act in different ways in cell lines, possibly due to the different levels of aggressiveness of the tested strains [73].

Similar to this study, Giraulo et al. observed different modulations in the A375 and SK-MEL-28 cell lines [74], in which they observed a greater increase in purinergic signaling components and inflammatory pathways after nutrient deprivation in the A375 cell line, whereas in the SK-MEL-28 cell line, basal levels were maintained, indicating that the different cell behavior is due to differences in aggressiveness and metastatic potential.

The divergent CD39/CD73 responses between A375 and SK-MEL-28 deserve a more nuanced interpretation. In A375 cells, the increase in CD39/CD73 transcripts at 6.25 µg/mL appears at first sight inconsistent with the proposed anti-immune-evasion narrative, since increased CD39/CD73 activity typically enhances extracellular adenosine production and immunosuppression. Several non-mutually exclusive explanations may account for this observation. (i) Stress-induced upregulation: CD39 and CD73 are induced in cells under metabolic and apoptotic stress, where they participate in the conversion of pro-inflammatory eATP into immunosuppressive adenosine as part of an acute stress response [75]. The concomitant increase in TNF-α, IL-6, and NLRP3 transcripts in A375 cells indicates that the cells are under stress at this concentration, which may, in turn, trigger compensatory induction of CD39/CD73. (ii) Functional vs. transcriptional discordance: Although CD39 and CD73 mRNA increased in A375, AMP hydrolysis (the CD73 functional readout) did not change significantly, suggesting that the transcriptional upregulation does not translate into proportional enzymatic activity. This dissociation between mRNA and function is consistent with a stress-coupled response that does not necessarily produce the immunosuppressive output predicted by the steady-state model. (iii) Cell-line-specific basal phenotype: A375 (high-MAGS, BRAF V600E) and SK-MEL-28 (low-MAGS) display markedly different baseline expression of cytokines, MMPs, and ectonucleotidases [45], and the regulation of CD73 in A375 has been specifically linked to TGF-β1 induction under stress conditions [74]. The differential response to AC3^®^, therefore, likely reflects an interaction between treatment and the intrinsic transcriptional program of each cell line. These observations are hypothesis-generating and require validation in additional cell lines, including patient-derived primary melanoma cells stratified by aggressiveness markers (BRAF status, PTEN loss, MITF levels), as well as protein-level and functional assays to confirm whether the observed transcriptional changes translate into modulation of adenosine production in the tumor microenvironment.

Lower extracellular adenosine levels, due to reduced CD73 expression, can reduce immunosuppression, thereby increasing the response to therapies [76]. One of the therapies for treating cutaneous melanoma with the highest levels of efficacy inhibits an immune checkpoint via PD-1/PD-L1 blockade [77]. However, high resistance rates persist, so recent studies support the use of associated therapies [78]. This association is observed in the study by Tu et al., who found that the combination of anti-PD-L1 and anti-CD73 antibodies improved treatment response in non-small cell lung cancer [79].

In melanoma, we observed that treatment with AC3^®^ decreased CD73 expression in SK-MEL-28 cells. This agrees with our previous study, in which CUR exerted similar activity in SK-MEL-28 cells. Thus, both CUR and the curcuminoid mixture are strong candidates for adjuvant therapy of CM. One limitation of the study was that it did not evaluate the parameters of each substance individually. We intend to validate this in future studies. This study, however, was conducted entirely in vitro, and therefore, we suggest further studies evaluating the antineoplastic activity of these compounds *in vivo.*

Limitations of the study. Several limitations of the present work should be acknowledged. First, the mechanistic conclusions are largely based on transcriptional data (RT-qPCR), and protein-level validation by Western blot (cleaved caspase-3, cleaved caspase-8, TNFR1, CD39, CD73) and functional assays (caspase enzymatic activity, TNF-neutralizing experiments, ROS scavenger experiments) will be required to firmly establish the activation of the proposed pathways. Therefore, claims regarding modulation of the TNF/TNFR1 axis and inflammasome regulation should be interpreted as transcriptional associations rather than as evidence of functional pathway activation. Second, the wound-healing assay was performed without mitomycin C pre-treatment to inhibit proliferation; although the dissociation between concentrations affecting viability and migration (particularly the migration inhibition observed at 1.56 µg/mL in A375 in the absence of significant cytotoxicity) supports a genuine anti-migratory effect, future work should employ proliferation-controlled migration assays (e.g., transwell with mitomycin C). Third, this study did not include non-malignant control cells (e.g., primary human epidermal melanocytes or HaCaT keratinocytes); therefore, conclusions regarding tumor selectivity cannot be drawn from the present in vitro data, although the favorable safety profile of AC3^®^ has been previously documented in vivo [58]. Fourth, NLRP3 transcript levels reflect inflammasome priming (signal 1) but not assembly or activation; ASC speck formation, caspase-1 cleavage, and IL-1β/IL-18 maturation assays were not performed. Fifth, the translational implications discussed here, particularly with respect to potential synergy with immune checkpoint inhibitors (anti-PD-1/PD-L1), are speculative and require co-culture experiments with peripheral blood mononuclear cells (PBMCs) or tumor-infiltrating lymphocytes (TILs), measurement of PD-1/PD-L1 surface expression, and ultimately in vivo validation [80]. Sixth, single-substance comparisons (CUR, DMC, BDMC alone vs. the AC3^®^ mixture at equimolar concentrations) were not performed in this study and are planned for follow-up work to formally establish synergistic activity. These limitations frame the present findings as hypothesis-generating in vitro evidence that warrants further mechanistic and translational investigation.

## Conclusions

In summary, the present in vitro study demonstrates that AC3^®^ reduces cell viability, inhibits cell migration, decreases reactive oxygen species levels, and modulates the gene expression of pro-apoptotic markers (caspase-3 and caspase-8), pro-inflammatory mediators (TNF-α, IL-6, NLRP3), and components of the purinergic system (CD39, CD73) in two cutaneous melanoma cell lines, A375 and SK-MEL-28. Importantly, the responses observed differ between the two cell lines, likely reflecting their distinct aggressiveness profiles and intrinsic transcriptional programs. The findings reported here are based predominantly on transcriptional data and should therefore be interpreted as evidence of pathway-level associations rather than as evidence of functionally active pathways. Protein-level validation (Western blot for cleaved caspases, TNFR1, CD39, CD73), functional assays (caspase enzymatic activity, TNF-neutralizing experiments, ROS scavengers), inclusion of non-malignant control cells, proliferation-controlled migration assays, and ultimately in vivo studies will be required to confirm the mechanisms proposed and to evaluate translational relevance, including potential combination strategies with immune checkpoint inhibitors. Within these limitations, AC3^®^ is an *in vitro* candidate warranting further preclinical investigation as a potential adjuvant in cutaneous melanoma research.

## Supplementary Information

Below is the link to the electronic supplementary material.

Supplementary Material 1

## Data Availability

All data are available in the main text or supplementary materials.

## Declarations

### Declaration of IA use

During the preparation of this work, the authors used the tool Claude, version Opus 4.8, developed by Anthropic, for grammar review and restructuring of ideas. After using this tool, the authors reviewed and edited the content as needed and take full responsibility for the final content of the publication.
