# Supplementary material for: AC3® exerts cytotoxic and anti-migratory activity and modulates the gene expression of TNF-α, inflammatory mediators, and components of the CD39/CD73/adenosine axis in cutaneous melanoma cell lines
Source: Med Oncol. 2026 Jul 15;43(9):221. doi: 10.1007/s12032-026-03321-7 (PMC13372914; doi:10.1007/s12032-026-03321-7)
Supplement: Supplementary file 1 — Supplementary Material 1 [file 12032_2026_3321_MOESM1_ESM.pdf]

SABINSA CORPORATION

***Pharmaceuticals** ***Phytochemicals**
***Fine Chemicals** ***Herbal Extracts**
***Cosmeceuticals** ***Specialty Chemicals**

CERTIFICATE OF ANALYSIS

**Product Name**	**CURCUMIN AC3 COMPLEX (CURCUMA LONGA EXTRACT)**
**Product Code**	**0353**
**Batch No.**	**C230334**
**TR No.**	**KL23F0125**
**Date of Manufacture**	**February 2023**
**Date of Expiry**	**January 2028**

Category	Intended for Nutraceutical application
Botanical/Scientific name	Curcuma longa
CAS No	84775-52-0
Plant part	Rhizomes
Preparation type	Extraction
Solvent used for extraction	Ethyl acetate
Solvent used in manufacture	Ethanol
Final extract ratio	30:1 to 32:1
Standardization	Total Curcuminoids
Excipient used	None

**Parameters**	**Result**	**Limit**	**Reference**
**PHYSICAL**			
Description	Complies	Orange yellow powder	Visual
Identification	Complies	To comply by HPLC	SLL/STP-C-103
Loss on drying	0.47 % w/w	Not more than 2.0% w/w (dried at 105°C)	USP <731>
Ash Content	0.11 % w/w	Not more than 1.0% w/w	USP <561>
Tapped bulk density	0.67 g/ml	Between 0.50g/ml and 0.90g/ml	USP <616>
Loose bulk density	0.38 g/ml	Between 0.30g/ml and 0.50g/ml	USP <616>
Sieve Test (Passes Through)			USP <786>
- 20 Mesh	100.00 % w/w	Not less than 95% w/w	
- 40 Mesh	100.00 % w/w	Not less than 75% w/w	
- 80 Mesh	97.99 % w/w	Record	
**CHEMICAL**			
Assay			

SABINSA CORPORATION

*Pharmaceuticals *Phytochemicals
*Fine Chemicals *Herbal Extracts
*Cosmeceuticals *Specialty Chemicals

CERTIFICATE OF ANALYSIS

**Product Name**	**CURCUMIN AC3 COMPLEX (CURCUMA LONGA EXTRACT)**
**Product Code**	**0353**
**Batch No.**	**C230334**
**TR No.**	**KL23F0125**
**Date of Manufacture**	**February 2023**
**Date of Expiry**	**January 2028**

**Parameters**	**Result**	**Limit**	**Reference**
-Content of total curcuminoids by HPLC	86.08 % w/w	Not less than 85.0% w/w and not more than 95.0% w/w on dry basis	SLL/STP-C-103
Purity by HPLC			
-Bisdemethoxycurcumin	35.76 %	Not less than 30.0% and not more than 40.0%	SLL/STP-C-103
-Demethoxycurcumin	15.36 %	Not less than 15.0% and not more than 25.0%	SLL/STP-C-103
-Curcumin	48.88 %	Not less than 40.0% and not more than 50.0%	SLL/STP-C-103
**OTHERS**			
Lead	<0.2 ppm (µg/g)	Not more than 2ppm (µg/g)	USP <2232>
Arsenic	<0.2 ppm (µg/g)	Not more than 1ppm (µg/g)	USP <2232>
Cadmium	<0.2 ppm (µg/g)	Not more than 1ppm (µg/g)	USP <2232>
Mercury	<0.02 ppm (µg/g)	Not more than 0.1ppm (µg/g)	USP <2232>
Residual solvents	Complies	To comply as per USP	USP <467>
Residual pesticides	Complies	To comply as per USP	USP <561>
Glyphosate	Complies	Not more than 7ppm	SLL/STP-P-122
Total aflatoxins (B1,B2,G1 and G2)	Complies	Not more than 10ppb (µg/kg)	SLL/STP-A-044
-Aflatoxin B1	Complies	Not more than 5ppb (µg/kg)	SLL/STP-A-044
**MICROBIAL**			
Total aerobic microbial count	<100 cfu/g	Not more than 5000cfu/g	USP <2021>
Total yeasts and molds count	<10 cfu/g	Not more than 100cfu/g	USP <2021>

**Product Name**	**CURCUMIN AC3 COMPLEX (CURCUMA LONGA EXTRACT)**
**Product Code**	**0353**
**Batch No.**	**C230334**
**TR No.**	**KL23F0125**
**Date of Manufacture**	**February 2023**
**Date of Expiry**	**January 2028**

**Parameters**	**Result**	**Limit**	**Reference**
Escherichia coli	Complies	Negative/10g	USP <2022>
Salmonella	Complies	Negative/10g	USP <2022>
Staphylococcus aureus	Complies	Negative/10g	USP <2022>
Pseudomonas aeruginosa	Complies	Negative/10g	USP <62>
Bile tolerant gram negative bacteria	Complies	Negative/10g	USP <2021>
Coliforms	<10 cfu/g	Less than 10cfu/g	BAM 2001, 8th Edition, Chapter 4

ADDITIONAL INFORMATION

Sanitising treatment	Non irradiated and not treated with ETO
Certification status (Kosher/Halal)	Kosher and Halal certified
BSE/TSE status	BSE/TSE free
Genetic modification status	Non-GMO Project Verified
Country of origin	India
Cultivated or wild crafted	Cultivated
Storage condition	Store at room temperature
Manufactured By	Sami-Sabinsa Group Limited - Peenya<br>19/1, 19/2, I Main II Phase, Peenya Industrial Area, Bangalore. 560 058, Karnataka, India
Manufactured At	Sami-Sabinsa Group Limited - Peenya<br>19/1, 19/2, I Main II Phase, Peenya Industrial Area, Bangalore. 560 058, Karnataka, India
Remarks	*Since it is a herbal product, there is likely to be minor color variation from batch to batch of the product<br>because of the geographical and seasonal variations of the raw material.<br>*Natural products can be hygroscopic and may agglomerate sometimes. It is suggested to sift the product before use.

Product Name	CURCUMIN AC3 COMPLEX (CURCUMA LONGA EXTRACT)
Product Code	0353
Batch No.	C230334
TR No.	KL23F0125
Date of Manufacture	February 2023
Date of Expiry	January 2028

Dr. Hari Ramachandran
QA/ QC Manager

The above certificate of analysis is based on ***Specifications Issue No: 1 Dated: August 14, 2021***

NAP:Not Applicable
NAV:Not Available
